# The ‘Other’ face of health security: Reimagining epistemic attachments in global health

**DOI:** 10.1371/journal.pgph.0007105

**Published:** 2026-08-14

**Authors:** Max D. López Toledano, Mervat Alhaffar, Ali Alshalah, Majd Saleh, Anna Durrance-Bagale, Charnele Nunes, Esther Sharma, Natasha Howard

**Affiliations:** 1 Saw Swee Hock School of Public Health, National University of Singapore and National University Health System, Singapore, Singapore; 2 Department of Global Health & Development, London School of Hygiene and Tropical Medicine, London, United Kingdom; 3 Communicable Diseases Control Centre, Baghdad, Iraq; 4 King’s College London, Methodologies Division, Strand, London, United Kingdom; Institute of Tropical Medicine: Instituut voor Tropische Geneeskunde, BELGIUM

## Abstract

The ‘health security’ paradigm has reshaped global health since Covid-19, framing infectious diseases as existential threats and targeting marginalised populations as risky ‘others’ to surveil and contain. However, its exclusionary consequences for those ‘othered’ remain underexamined, particularly from the perspective of lived experience. Drawing on ethnographic and archival case studies in Lebanon, Nepal, Serbia, and Syria (2021–2025), we examine health security’s ‘other’ face, the systematic exclusion of migrant, rural, and displaced populations from protection, ranging from vaccine apartheid to medicalised border controls. We argue that these are not abstract harms but embodied burdens, and that ethnographic perspectives are essential to reveal them. Rejecting the premise that securitisation as the only available logic, we propose five cooperative mechanisms, including demilitarised financing, a binding vaccine convention, and data sovereignty, to shift global health toward equity and care. The question is not whether we can imagine different futures, but whether we have the courage to build them.

“*Even if we cannot deny that some problems are unprecedented and some solutions are innovative, it could be that global health is less about new problems (or solutions) than about new “problematizations,” to use Michel Foucault’s term; that is, new ways of describing and interpreting the world — and therefore of transforming it*.”.

Didier Fassin, 2012

The notion of ‘health security’ has emerged in the last three decades to describe global shifts in dominant approaches to public health [1]. This notion—often applied as ‘global health security’—locates aspects of public health within a global ‘security’ political architecture, framing health concerns as direct threats to the nation-state. Its defining characteristics include the depiction of diseases as political ‘enemies’ [2], redirection of resources from health systems to emergency responses [3,4], a focus on infectious diseases and ‘pandemic preparedness’ [5,6], and involvement of military actors in public health services provision [7]. Scholars across disciplines and geographies have reported on the involvement of military institutions in health security activities as the pinnacle of an ongoing “normalization of military activities other than war” (e.g., [8–11]). While the relationship between health and militarism is complex and warrants further unpacking, the convergence of these sectors has been empirically documented and critiqued.

Following the Covid-19 pandemic (2020–2023), the world entered a ‘post-pandemic’ era anchored by global health security as a medico-cultural pillar. That is, a social, political, scientific, and medical landscape in which the ideological tenets of health security have become dominant features in the way we think and understand the world, both in popular culture and technical disciplines. Importantly, we employ ‘post-’ here as political theorist Wendy Brown does, referring to “a very particular condition of afterness in which what is past is not left behind, but, on the contrary, relentlessly conditions, even dominates, a present that nevertheless also breaks in some way with this past” [12]. We thus do not consider this ‘post-pandemic’ era as one in which Covid-19 no longer exists, but rather as one that is “temporally after but not over” its global spread [12]. The uncertainties of 2020 have been routinised as a “new normal,” and the exclusions that took place during its epoch remain. Namely, Covid-19 sedimented health security as a socio-political paradigm rooted in various forms of exclusion, surveillance, and social division.

Consequently, even amidst calls to incorporate equity initiatives into health security (e.g., [13]), critical scholars have insisted on making its irrevocable ‘neo-colonial’ character explicit (e.g., [14]). The result has been a blurred understanding of what health security tangibly means. McCoy and colleagues [15] note ‘health security’ has proliferated as a catch-all term, from advocating for universal healthcare access to implying the incorporation of public health into what anthropologist Catherine Besteman calls the “global security-industrial complex” [16]. Such polyvalence is paradoxical. ‘Security’ invokes and depends on the management, exclusion, and elimination of the risky ‘others’ it protects against, while health ‘equity’ is “achieved when everyone can attain their full potential for health and well-being” [17]. Both cannot be accomplished in tandem because they contradict each other.

Our research therefore situates health security as a driving force of exclusion in the name of public health, and one whose conceptual vagueness allows it to be wielded for political ends. Specifically, by inviting a relationship with securitised discourse, (global) public health becomes a vehicle of geopolitical power through which a “state of exception” may be declared and maintained [18,19]. In such situation, a juridical and political “ambiguous zone” that stems from a declared state of emergency [18, 2], rights are withdrawn and political formations reshaped. And most importantly, as political theorist Achille Mbembe writes in *Necropolitics*, the state of exception cultivates a “perception of the existence of ‘the Other’ as an attempt on my life, as a mortal threat or absolute danger whose biophysical elimination would strengthen my life potential…” [20]. It exacerbates —if not creates— the socio-political vulnerabilities that marginalized communities struggle with, justifying them as merely part of “the activities required, both proactive and reactive, to minimize the danger and impact of acute public health events that endanger people’s health across geographical regions and international boundaries”, as per the [21] definition of health security. In other words, it takes living communities as political sacrifices, disguising this process as public health.

Certainly, these lines of thinking have been consistently articulated by critical perspectives in public health, medical humanities, and the social sciences (e.g., [14,15,19,22,23]). However, beyond critique, our invitation is to pay attention to an understanding of health security and its effects that does not treat these as purely theoretical dangers, disembodied ailments, statistical trivia, or cautionary tales, as these have frequently been handled. Rather, borrowing from the discipline of cultural anthropology, our call is to interrogate what health security is when we think of it from the vantage point of lived experience. This lived experience refers not just to our own (though investigating our location in relation to systems of power is always crucial work) but especially to the lived realities of those who are targeted as the ‘others’ of securitised views of health. Such ‘others’ have a face. Our duty as global health scholars is to look at them and listen to what they have to say directly. We might learn a lot.

Focused on the Asia region, this article aims to consider who these ‘others’ are and what health security might look like from their perspectives. From this standpoint, we defined our research question as: ‘What are health security’s foundations and attachments, what lessons might it teach us for the present and future of global health, and what alternatives can we envision?’

## Methodology

We re-analysed findings from ethnographic and archival case studies (2021–2025) conducted across our research themes on expressions of health security in disease surveillance, vaccine geopolitics, medicalised border tightening, and neglected environmental health issues. While these cases are distinct in their geographic and thematic contexts, they are connected by the presence of health security logics and our analytical focus on how research participants experienced, resisted, or made sense of these logics. This research was conducted with differentially marginalised communities shaped or affected by health security in Nepal, Syria, Lebanon, and the Balkans [6,24–29]. All studies were originally conducted without focusing on health security. Our re-analysis thus focused not on health outcomes, policy priority-setting, or production of epidemiological statistics, but instead on how health security initiatives intersect with these global health topics and the efforts by research participants to make sense of the health interventions they are subjects of, at times framed as being in their benefit, at times explicitly excluding them. We asked, for example, “in what forms are the experiences described linked to health security initiatives, how do participants describe these, or what alternative forms of public health work, if any, are suggested formally or informally by research participants?”

In so doing, we collated a new perspective on what health security is, implies, and does, re-constructed by piecing together the experiences of those at its margins, the policy architecture it relies on, and tracing the funding streams that bring it together. Integrating this, we put forward the notion of the “Other” face of health security, simultaneously alluding to the *othering* processes that cast marginalised communities as threatening, pathological, excludable, or somehow less worthy of protection, and to the underside of health security that official global health messaging obscures yet which becomes apparent when tracing its phenomenological consequences.

## The ‘other’ face of health security

If ‘security’ is ultimately a quest to “identify and contain ‘risky’ people” [16], tracing epidemiological ‘risk’ narratives should reveal health security’s ideological commitments. This is not a difficult task, tractable from the way global health security funding streams have proliferated [30] to how the Covid-19 pandemic exposed which populations can expect to be first sacrificed. Migrant populations, especially those displaced and navigating already restrictive mobility regimes, took the biggest hit. After all, across the world, refugees are framed as existential threats. Our research with Syrian refugees in Lebanon found national media depicting them as “lacking healthy and safe, germ-free conditions” and threatening the “Lebanese health body” [31,32], intertwining health concerns with the enforcement of cultural borders. Similarly, a recurrent theme in our research with undocumented Afghan women migrating to Europe through the Balkans showed that they were subject to prolonged military-enforced quarantine and restricted access to basic health services [29]. Across these contexts, implementation of ‘sanitary passports,’ as historian of science Joelle Abi-Rached [33] describes, reinforced divisions between bodies deemed secure and insecure. In both cases, *who* health security worked to exclude was poorly veiled.

Comparably, disease surveillance has expanded and become a priority, as ambitions of ending the ‘next pandemic’ before it begins become central to the global health security agenda. Global health funding now largely prioritises pre-emption of pandemic risk over provision of services to address existent health needs [34]. These efforts, however, target ‘global South,’ rural, agricultural, and Indigenous populations labelled as risks and potential conduits for an emergent infectious disease (EID) spillover [6,35]. Social scientists (e.g., [36,37]) note that such risk constructions are epistemologically based on speculation and even prophecy, while being susceptible to political manipulation. As we reported through our ethnographic research from Nepal [6], such speculation allows social, cultural, and political anxieties to be weaponised. More specifically, they align with what Alexandre [38, 26] terms “epidemic orientalism”, an othering process through which infectious disease governance acts as a “vehicle for ascribing difference geographically, racially, ethnically, and nationally”. Prejudice towards already-marginalised populations is amplified by an emphasis on the risk management of hypothetical infectious disease, whereas existent environmental health vulnerabilities and non-communicable disease risks remain largely ignored [27].

This pattern illustrates what we term the funding paradox, with populations labelled as ‘risky’ attracting surveillance and risk communication resources yet remaining systematically under-resourced for basic health services. We argue this paradox is structural, emerging from the very logic of health security that treats these populations primarily as potential disease conduits rather than as people with health needs.

For these populations, at the margins of this securitised landscape, global health narratives and initiatives can appear cynical. The ‘vaccine apartheid’ during the Covid-19 pandemic did little to help, demonstrating sacrificial logics. Six months after public rollout, approximately 80% of four billion available doses went to high-income countries [39] causing at least one million preventable deaths in low and middle-income countries (LMICs) [40]. That the people excluded from access to life-saving medical technologies would divest from global health efforts is no surprise. For example, our participants in rural areas of Nepal targeted for disease surveillance investments displayed relative indifference to risk awareness campaigns [6,41]. Simplistically-designated ‘beneficiaries’ recognise they are not the ultimate beneficiaries, for global health as a field is out of touch with their lived realities and its interventions are primarily to protect *others elsewhere*. At best, they are framed as recipients of this or that ‘Western/Global North’ intervention, but asymmetries in who is involved in decision-making, or even has their story heard, have been widely documented (e.g., [42–44]).

Across studies, we found a consistent pattern of neglect, de-prioritisation, and active framing of marginalised and global South populations as risky subjects, informing how people perceived themselves and their relationship to global health. In most cases, the global narratives of securitised health clashed with an understanding by individuals of their lives in vulnerable social conditions. Paradoxically, the communities most exposed to zoonotic disease emergence were among the least likely to receive adequate treatment, yet the most likely to be targets of risk awareness campaigns [6]. The communities facing most compound vulnerabilities from forced displacement, unhospitable host societies, and poor living conditions in camps were also subject to military containment, deemed a risk to manage rather than humans to care for [29]. These are but some examples, with the same patterns replicated across the world. It is thus unsurprising that some communities would show relative indifference towards Covid-19, making narratives that framed the pandemic as “just another way to die” relatively common [26]. The infectious disease that, for a global health regime shaped by securitised narratives, became a prioritised organising pillar was for some an addendum to the many issues they already faced.

Global health initiatives, if existent, do little to “save” communities that define their issues on different terms. Securitisation policies worsen this relationship, fortifying a wall between global health and the people it supposedly cares for. To call for a global health security “for all,” in that sense, is beyond paradoxical. From an ethnographic view that centres the lived experience of “recipients,” health security is a farcical effort to disguise a sacrificial ideology that has long given up on certain populations. It is, indeed, a two-faced regime. One face claims to ensure the health of society—*both proactively and reactively*—, whereas the other makes sure such society is narrowly defined. One face enjoys protection through ever more advanced technologies and military resources, whereas the other is continuously surveilled and contained via the same means. There is health security, and then there is its other face.

## From critique to alternatives

Our analysis is not a damning sentence on global health. It points at a historically contingent nexus between global health and security sectors—a colonial epistemic paradigm held together by untenable ethical and political premises. It need not be final. It can—and should—be replaced with new knowledge foundations attuned to a broader range of risks and based on principles of equity and care across political borders, socio-cultural strata, and planetary ecology.

Abandoning militaristic public health approaches and vocabulary is the most direct route out of the colonial landscape in which global health originates, yet deep ideological transformation must occur before equity and justice can be approximated. Health equity approaches that centre “dignity” need to be embraced [45], whereas instruments designed to monitor unjust practices in the production of global health knowledge must continue to be promoted (e.g., [46]). After all, abandoning the notion that we can simply shield ourselves by force and push risks to others is to acknowledge our shared responsibilities in today’s interconnected world. The securitised view is not cause but symptom of entrenched beliefs that justify survivalist attitudes at the expense of others. As Fassin argues, global health is guided not only by measurable ‘truth’ but by polyvalent and subjective meaning and can therefore be (re)shaped [47].

Drawing on our empirical findings (Table 1), we propose five aspirational mechanisms (Table 2), grounded in documented failures and potential solutions articulated within affected communities. As global health professionals, we must ask what values our ideas reproduce implicitly and explicitly, who are we accountable to, and how we are serving (or not) the people our field has long claimed to value yet leaves at its margins. In doing so, we must promote, and actively seek, collaboration across boundaries—political, cultural, disciplinary—including with those sidelined at the margins.

**Table 1:** Two opposing global health paradigms.

Dimensions	Security paradigm (current)	Cooperative paradigm (potential)
Core logics	Containment of risky ‘others;’ sacrifice of marginalised populations to protect the privileged	Shared responsibilities across borders; recognition of mutual vulnerability
Key actors	Military, surveillance agencies, high-income country (HIC) governments	HIC-LMIC coalitions, epistemic communities, mutual aid networks
Resource flows	Emergency response → HIC biosecurity	Routine systems strengthening → locally governed priorities
Intervention targets	High-risk infectious diseases, EID ‘hotspots’	Eco/planetary health, social determinants/NCDs, high burden infectious diseases
Marginalised populations	Excluded, surveilled, sacrificed	Included in governance, benefits-sharing, data ownership
Accountability	Top-down (HICs, WHO) with limited mechanisms for enforcement	Horizontal (treaty enforcement, localised stewardship, community data sovereignty)

**Table 2:** Shifting from securitisation to cooperative care logics.

Mechanism	Potential approach	Findings	Sources
1. Demilitarised financing	Redirect a minimum % of global military expenditure (e.g., 15% or $300 billion annually) to a Global Health Commons supporting conflict-affected and LMIC health systems.	Conflicts destroy lives and damage/fragment health systems. Militarised health spending diverts resources from routine systems into securitised responses that harm equity and health outcomes.	[24,26]
2. Vaccine convention	A binding WHO Framework Convention on Pandemic Product Equity with automatic technology transfer within 90 days of emergency use authorisation, binding price caps based on tiered pricing, and distributed regional manufacturing with technology transfer.	Technology transfer was blocked; voluntary mechanisms (e.g., COVAX) failed to achieve equity.	[25,48,49]
3. Data sovereignty	Establish community health data trusts (e.g., adapted from Indigenous data sovereignty initiatives in Australia and Canada) that give communities veto power over which EID risks are surveilled; mandate that a minimum of 30% of surveillance funding be directed to locally prioritised health programmes.	Surveillance without consent generates rational indifference; data used for exclusion rather than care.	[6,24]
4. Risk framing	Mandate a minimum % of pandemic funding addresses environmental/planetary health and NCDs, establishing parity with infectious disease funding in LMIC contexts.	Environmental health and NCDs are leading burdens but receive <2% of emergency funding.	[27,28,43]
5. Cooperation Index	Replace the Global Health Security Index with a Cooperation Index ranking countries on cross-border resource sharing (e.g., vaccine donations, technology/data transfer, health worker deployment), technology transfer (e.g., number and speed of voluntary or compulsory licences), refugee health access (e.g., right to care, non-discrimination).	Security indices reward isolation, informal cross-border cooperation is necessary and can be effective but is unfunded.	[26,29]

In this spirit, we must also contend with the political economy interests that sustain the security paradigm. As Robertson et al. [30] demonstrate, funding for global health security has increased dramatically, creating institutional path dependencies that resist reform. Recognising these material interests is essential for any credible strategy to transition toward cooperative approaches.

Geopolitical polarisation within a multipolar world has already demonstrated its necessary consequences: the preventable deaths of millions due to self-interested ideologies (in both Global North and South) that see most people as necessary sacrifices, dehumanized Others, for the survival of a wealthy few. The COVID-19 pandemic demonstrated that such polarisation is not a geopolitical abstraction but a concrete determinant of life and death. At least one million preventable deaths in LMICs resulted from vaccine hoarding by high-income countries [40]. This statistic is not an indictment of any single nation but *is* symptomatic of a global system that normalises sacrifice.

Accordingly, we must continually revisit our cultural attitudes within the field, and we must listen deeply to those who have been left on the margins thus far. Therein lie our most valuable epistemic attachments. We need to (re)learn who the people we serve are—not as data points, but *as people*. Can we speak their languages? Are we letting manufactured crises interfere with ethical duty? Most importantly, do we dare to care beyond biomedical intervention, questioning the transnational architectures of power that create health asymmetries in the first place?

## Conclusion

Health security’s ‘Other’ face reveals systematic exclusion of Global South populations and Global North/West sub-populations, treated as risks to contain rather than people to protect. This exclusion is a constitutive feature of the security paradigm, one that redistributes life chances in favour of the already privileged. Alternatives exist, but require minimising the security paradigm’s seductive urgency and instead building accountability mechanisms, data sovereignty frameworks, and inclusive care infrastructures. These may sound like utopian ideals but are actually pragmatic adaptations of models already tested in Indigenous data sovereignty initiatives, cooperative pandemic response networks, and community-led health programmes. Health professionals globally are uniquely positioned to advocate for this shift, not by abandoning technical expertise, but by recalibrating whose knowledge counts and whose lives matter. The question is not whether we can imagine a different future, but whether we have the courage to build it.

### Highlights

What is already known:

- ‘Health security’ has become a dominant global health paradigm, framing infectious diseases as existential threats and justifying securitised responses that prioritise containment over care.

What this article adds:

- An ethnographic account of health security’s ‘other’ face: how marginalised populations are systematically excluded, treated as threats, or sacrificed, and a call for methodological innovation by centring lived experience to reveal these embodied harms.

Policy implications:

- Replacing militaristic approaches with cooperative mechanisms, including demilitarised financing, a binding vaccine convention, and community data sovereignty, could help shift global health from exclusion to equity.
